# Premenstrual Symptoms in Dysmenorrheic College Students: Prevalence and Relation to Vitamin D and Parathyroid Hormone Levels

**DOI:** 10.3390/ijerph9114210

**Published:** 2012-11-16

**Authors:** Bayan A. Obeidat, Haifa A. Alchalabi, Khalid K. Abdul-Razzak, Mudhaffar I. Al-Farras

**Affiliations:** 1 Department of Nutrition and Food Technology, Faculty of Agriculture, Jordan University of Science and Technology; P.O. Box 3030, Irbid 22110, Jordan; 2 Department of Obstetrics and Gynecology, Faculty of Medicine, Jordan University of Science and Technology, P.O. Box 3030, Irbid 22110, Jordan; Email: halchalabi@yahoo.com; 3 Department of Clinical Pharmacy, Faculty of Pharmacy, Jordan University of Science and Technology, P.O. Box 3030, Irbid 22110, Jordan; Email: kkalani@just.edu.jo; 4 Department of Emergency, King Abdullah University Hospital, Irbid 22110, Jordan

**Keywords:** premenstrual symptoms, primary dysmenorrheal, vitamin D, parathyroid hormone, dairy products

## Abstract

*Objectives:* To determine the prevalence of premenstrual symptoms (PMS) due to primary dysmenorrhea among a sample of university female students, and to explore possible association with vitamin D and parathyroid (PTH) levels, as well as frequency of consumption of dairy products. *Design: *A cross-sectional study. *Setting: *One Jordanian university. *Subjects: *A total of 177 female students aged between 18 and 24 years who experienced primary dysmenorrhea participated in the study and completed a self administered questionnaire to collect information concerning demographics, menstruation- related information, associated specified premenstrual symptoms, and consumption of dairy products. Plasma 25-hydroxyvitamin vitamin D level and intact parathyroid hormone level were measured. *Results*: Of the 177 participants 91.5% had two or more symptoms among which fatigue, mood swings, anxiety, abdominal bloating, and depression were the most prevalent symptoms. There was no evident association between presence of symptoms and vitamin D status, PTH level or dairy products consumption. Headaches and social withdrawal were significantly lower in those women who consumed high amounts of dairy products. *Conclusion: *Premenstrual symptoms are very common in young women with primary dysmenorrhea. PMS has no relation to levels of vitamin D, parathyroid hormone or dairy products consumption. Headache and social withdrawal may be affected by dairy product consumption.

## 1. Introduction

Premenstrual symptoms, often collectively referred to as premenstrual syndrome (PMS), and dysmenorrhea, are common gynecological problems affecting the lifestyle and performance of young women. Different types of studies from both developed and developing countries have found a consistently high prevalence of both dysmenorrhea and premenstrual symptoms in women of different ages and nationalities [1,2,3,4,5]. The majority of cases of dysmenorrhea are primary, which is defined as painful menses in women with normal pelvic anatomy that usually begins during adolescence [6]. Premenstrual syndrome (PMS)/premenstrual dysphoric disorder (PMDD) is defined as a group of disorders characterized by emotional, behavioral and physical symptoms that occur in the luteal phase of the menstrual cycle and subside following menstruation [7], but remain without definitive physical or laboratory criteria for diagnosis and are associated with some degree of inter-cycle variability [8]. Physical symptoms of this disorder include headaches, breast tenderness, abdominal bloating, peripheral edema and general fatigue, while psychological or behavioral disorders include irritability, mood swings, food cravings, social withdrawal, anxiety, and depression. While definitive diagnosis of these disorders remains debatable, a prospective record of cycle related symptoms is the gold standard for diagnosis by establishing a relationship between the symptoms and the late luteal phase of the menstrual cycle. Retrospective, self-reporting of symptoms is found to be reasonably sensitive [9,10]. 

The prevalence of PMS is variable [11]. Prevalence as high as 75–85% is mentioned if one or several symptoms is considered, 10–15% if medical care is requested and 2–5% with social activities interruption [12,13,14]. The causes of premenstrual symptoms are uncertain [15]. Many theories have been tested regarding possible causes of premenstrual symptoms. While many of the hormonal and biochemical profiles of women with premenstrual symptoms and those who are symptom free were similar, fluctuation in gonadal hormone levels may trigger the symptoms [16]. PMDD is thought to be related to serotonergic synapse abnormality and women with PMDD are found to have lower serotogenic function in the luteal phase [17,18]. A correlation between premenstrual symptoms and dysmenorrhea was made by Isaa and Tomko [19,20]. The severity of dysmenorrheal pain and premenstrual symptoms varies among women but it can be severe enough to cause a substantial negative impact on their daily activity. Current understanding of the pathogenesis in primary dysmenorrhea implicates excessive imbalanced amounts of prostanoids and possibly eicosanoids released from the endometrium [21,22]. The majority of subjects benefit from administration of nonsteroidal anti-inflammatory drugs (NSAIDs) [13,23]. A strong negative correlation between dairy product intake and dysmenorrhea and its associated symptoms among university female students was demonstrated whereby the severity of primary dysmenorrhea decreased with increasing daily intake of dairy products [24]. These results suggested that dietary calcium, among other substances, may have a functional role in the etiology of dysmenorrhea and a possible relation between calcium and vitamin D and premenstrual symptoms has also been suggested by Bertone-Johson and colleagues [25]. The aim of this study was to explore the prevalence of premenstrual symptoms in young college students known to suffer from with dysmenorrhea and to investigate any relation with vitamin D and parathyroid hormone levels or consumption of dairy products by these women.

## 2. Experimental Section

### 2.1. Participants and Setting

One hundred and seventy seven, single and healthy college students who experienced primary dysmenorrhea responded to our advertisement and accepted to take part in the study during the period from March to May 2010***.*** The Institutional Review Board Committee approved this study. Written informed consent was obtained from all participants. 

Participants with primary dysmenorrhoea were instructed to complete a guided self-assessment questionnaire. Information regarding participants’ age, age at menarch, menstrual cycle regularity, amount of blood loss and pain severity was collected. Blood loss is described as light (below average), moderate and heavy if associated with clots. Pain severity was graded as mild, severe and very severe as follows: *Mild*: pain that is tolerated without need for medications; *Severe*: pain that resolved with analgesics; *Very severe*: pain that is not relieved with analgesics and may interfere with usual daily activity.

Participants were also requested to answer questions about their recurrent experience of 12 symptoms during the premenstrual phase that subside with onset of menstruation. Psychological symptoms of irritability, anxiety, depression, mood swings, social withdraw, change in appetite and cravings for sweet or salty foods are recorded, along with physical symptoms such as abdominal bloating, headaches, general fatigue, nausea, and breast tenderness [26]. The study group included participants having a minimum of two symptoms, one physical symptom and one psychological symptom, while those with only one symptom or no symptoms acted as control. Assessment of dairy products intake was measured by number of specified portions consumed per day, per week or per month of any type of dairy products. Consumption frequency was calculated as times per day. BMI was calculated at the same time.

### 2.2. Blood Samples

About 10 mL of venous blood was collected from each participant in heparinized tubes. Plasma was assayed for plasma 25-hydroxyvitamin vitamin D [25(OH) D] level and intact parathyroid hormone (PTH) level. An enzyme immunoassay (The Immunodiagnostic System (IDS), UK) was used. The tests were performed in the central laboratory of Specialty Hospital (ISO 15189:2007)/Amman, Jordan.

### 2.3. Definitions

Vitamin D status was divided into three diagnostic categories according to plasma 25(OH)D levels. Vitamin D deficiency (VDD) <25 mmol/L, Vitamin D insufficiency (VDI) 25–75 mmol/L and Vitamin D sufficiency (VDS) >75mmol/L. Normal reference value of PTH is 13.9–75 pg/mL. These reference ranges were provided by the manufacturer of the assay.

### 2.4. Statistical Analysis

Data were analyzed using the Statistical Package for Social Science (SPSS, version 16.0). Descriptive statistics that include frequency and, range and mean with standard deviation were carried out. Chi-square (X^2^) test was performed to investigate the association between premenstrual symptoms with characteristics of participants and other variables of interest (vitamin D status, parathyroid hormone level, daily dairy product intake). Findings with *p* value <0.05 were considered to be statistically significant.

## 3. Results

**Figure 1:**
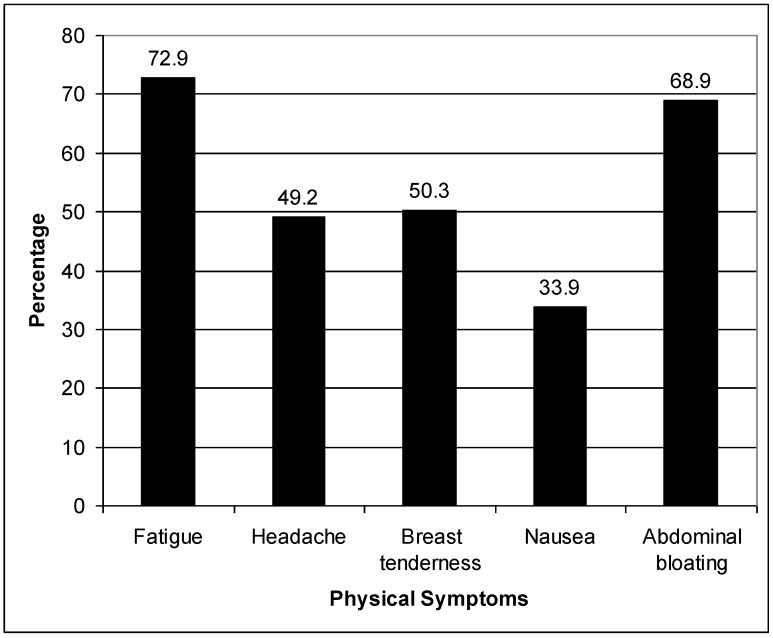
Distribution of physical symptoms among females with premenstrual syndrome.

**Figure 2:**
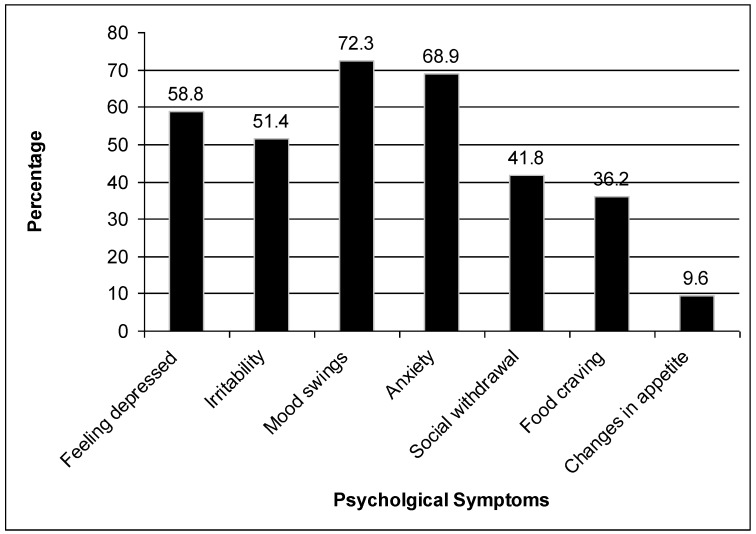
Distribution of psychological symptoms among females with premenstrual syndrome.

The age of the participants ranged between 18 and 24 years, with a mean of 21.8 ± 2.8 years. The mean body mass index value was 22.1 ± 1.1 Kg/m^2^. The mean age at menarche was 13.3 ± 1.4 years and dysmenorrhea started at an age of 15.3 ± 2.8. Approximately 17% of the participants had a menstrual cycle duration of less than five days, while 83% of them had menstrual cycle durations of five days or more. The majority of participants (77.8%) had regular menstrual cycles. Two or more premenstrual symptoms were detected in 91.5% of the participants. The most frequent symptoms are fatigue, mood swings, anxiety, abdominal bloating, and depression (Figure 1 and Figure 2). Approximately 22% of the participants graded their dysmenorrheal pain as mild, 68% as severe and only 9.6% of the participants reported very severe menstrual pain. Scoring of pain severity in relation to presence of premenstrual symptoms were highly significant (*p* value <0.001, Table 1). The relationship between premenstrual symptoms and vitamin D status, PTH level, daily dairy products intake was studied. No significant association between positive symptoms and vitamin D level, parathyroid hormone level or dairy products intake could be demonstrated (Table 2). Analysis of each symptom in relation to parathyroid hormone, Vitamin D and daily dairy products consumption did not reveal any significant association, apart from a negative association between dairy products intake and headache and social withdrawal (*p* value 0.036 and 0.044) respectively (Table 3, Table 4 and Table 5).

**Table 1:** PMS in relation to menstrual pattern.

Variable	Premenstrual symptomsn (%) ^a^		Total	*p value*
	No	yes		
Dysmenorrheal severity				<0.001
Mild	10 (25.6)	29 (74.4)	39	
Severe	5 (4.1)	116 (95.9)	121	
Very Severe	0 (0)	17 (100)	17	
**Regularity of menstruation**				0.39
Irregular	2 (5.1)	37 (94.9)	39	
Regular	13 (9.5)	124 (90.5)	137	
**Menstrual blood loss**				0.78
Light	1 (12.5)	7 (87.5)	8	
Moderate	13 (8.7)	136 (91.3)	149	
Heavy	1 (5)	19 (95)	20	

^a ^Data presented as numbers and percent within parenthesis.

**Table 2:** The association between parathyroid hormone, vitamin D, dairy products consumption and the risk of PMS.

Variable	Premenstrual symptoms n (%) ^a^		Total	*p value*
	No	yes		
Parathyroid hormone level ^b^				0.93
Normal	11 (8.6)	117 (91.4)	128	
Hyperparathyroid	4 (8.2)	45 (91.8)	49	
**Vitamin D level ^c^**				0.85
Normal	0 (0)	3 (100)	3	
Insufficiency	9 (9)	91 (91)	100	
Deficiency	6 (8.1)	68 (91.9)	74	
**Daily dairy products intake**				0.84
≤ 1 serving	3 (6.7)	42 (93.3)	45	
1–2 servings	9 (9.6)	85 (90.4)	94	
>2 servings	3 (7.9)	35 (92.1)	38	

^a ^Data presented as numbers and percent within parenthesis; **^b^** Normal reference value of PTH is 13.9–75 pg/mL, Hyperparathyroid: >75 pg/mL; **^c^** Vitamin D deficiency: <25 mmol/L, Vitamin D insufficiency: 25–75 mmol/L and Vitamin D sufficiency: >75mmol/L.

**Table 3:** Relation of symptoms to parathyroid hormone level.

Symptom	Parathyroid hormone status n (%) ^a^		*p value*
	Normal (n= 128)	Hyperparathyroidism (n= 49)	
Fatigue			
No	33 (68.8%)	15 (31.2%)	
Yes	95 (73.6%%)	34 (26.4%)	NS
Headache			
No	69 (76.7%)	21 (23.3%)	
Yes	59 (67.8%)	28 (32.2%)	NS
Breast tenderness			
No	66 (75.0%)	22 (25.0%)	
Yes	62 (69.7%)	27 (30.3%)	NS
Depression			
No	55 (75.3%)	18 (24.7%)	
Yes	73 (70.2%)	31 (29.8%)	NS
Irritability			
No	63 (73.3%)	23 (26.7%)	
Yes	65 (71.4%)	26 (28.6%)	NS
Mood swings			
No	38 (77.6%)	11 (22.4%)	
Yes	90 (70.3%)	38 (29.7%)	NS
Anxiety			
No	42 (76.4%)	13 (23.6%)	
Yes	86 (70.5%)	36 (29.5%)	NS
Social withdrawal			
No	78 (75.7%)	25 (24.3%)	
Yes	50 (67.6%)	24 (32.4%)	NS
Nausea			
No	84 (71.8%)	33 (28.2%)	
Yes	44 (73.3%)	16 (26.7%)	NS
Abdominal bloating			
No	40 (72.7%)	15 (27.3%)	
Yes	88 (72.1%)	34 (27.9%)	NS
Food craving			
No	83 (73.5%)	30 (26.5%)	
Yes	45 (70.3%)	19 (29.7%)	NS
Change in appetite			
No	13 (76.5%)	4 (23.5%)	
Yes	128 (72.3%)	49 (27.7%)	NS

^a ^Data presented as numbers and percent within parenthesis.

**Table 4:** Relation of symptoms to vitamin D levels.

Symptom	Vitamin D status n (%) ^a^			*p value*
	Deficiency (n = 74)	Insufficiency (n = 100)	Normal (n = 3)	
Fatigue				
No	18 (37.5%)	30 (62.5%)	0 (0%)	
Yes	56 (43.4%)	70 (54.3%)	3 (2.3%)	NS
Headache				
No	37 (41.1%)	53 (58.9%)		
Yes	37 (42.5%)	47 (54.0%)	3 (3.4%)	NS
Breast tenderness				
No	40 (45.5%)	48 (54.5%)		
Yes	34 (38.2%)	52 (58.4%)	3 (3.4%)	NS
Depression				
No	36 (49.3%)	36 (49.3%)	1 (1.4%)	
Yes	38 (36.5%)	64 (61.5%)	2 (1.9%)	NS
Irritability				
No	35 (40.7%)	50 (58.1%)	1 (1.2%)	
Yes	39 (42.9%)	50 (54.9%)	2 (2.2%)	NS
Mood swings				
No	17 (34.7%)	32 (65.3%)		
Yes	57 (44.5%)	68 (53.1%)	3 (2.3%)	NS
Anxiety				
No	19(34.5%)	36(65.5%)	0 (0%)	
Yes	55 (45.1%)	64 (52.5%)	3 (2.5%)	NS
Social withdrawal				
No	43 (41.7%)	60 (58.3%)	0 (0%)	
Yes	31 (41.9%)	40 (54.1%)	3 (4.1%)	NS
Nausea				
No	52 (44.4%)	64 (54.7%)	1 (0.9%)	
Yes	22 (36.7%)	36 (60%)	2 (3%)	NS
Abdominal bloating				
No	23 (41.8%)	31 (56.4%)	1 (1.8%)	
Yes	51 (41.8%)	69 (56.6%)	2 (1.6%)	NS
Food craving				
No	52 (46%)	60 (53.1%)	1 (0.9%)	
Yes	22 (34.4%)	40 (62.5%)	2 (3.1%)	NS
Change in appetite				
No	68 (42.5%)	89 (55.6%)	3 (1.9%)	
Yes	6(35.3%)	11(64.7%)	0 (0%)	NS

^a ^Data presented as numbers and percent within parenthesis.

**Table 5:** Relation of symptoms to dairy product intake.

Symptom	Daily dairy products consumption (serving) n (%) ^a^			*p value*
	≤1 (n = 45)	≤2 (n = 94)	>2 (n = 38)	
Fatigue				
No	11 (22.9%)	24 (50.0%)	13 (27.1%)	
Yes	34 (26.4%)	70 (54.3%)	25 (19.4%)	NS
Headache				
No	19 (21.1%)	46 (51.1%)	25 (27.8%)	
Yes	26 (29.9%)	48 (55.2%)	13 (14.9%)	0.036
Breast tenderness				
No	24(27.3%)	46 (52.3%)	18 (20.5)	
Yes	21(23.6%)	48(53.9%)	20(22.5%)	NS
Depression				
No	15 (20.5%)	40 (54.8%)	18 (24.7%)	
Yes	30 (28.8%)	54 (51.9%)	20 (19.2%)	NS
Irritability				
No	22 (25.6%)	44 (51.2%)	20 (23.3%)	
Yes	23 (25.3%)	50 (54.9%)	18 (19.8%)	NS
Mood swings				
No	13 (26.5%)	25 (51.0%)	11 (22.4%)	
Yes	32 (25.0%)	69 (53.9%)	27 (21.1%)	NS
Anxiety				
No	12 (21.8%)	28 (50.9%)	15 (27.3%)	
Yes	33 (27.0%)	66 (54.1%)	23 (18.9%)	NS
Social withdrawal				
No	19 (18.4%)	60 (58.3%)	24 (23.3%)	
Yes	26 (35.1%)	34 (45.9%)	14 (18.9%)	0.044
Nausea				
No	29 (24.8%)	66 (56.4%)	22 (18.8%)	
Yes	16 (26.7%)	28 (46.7%)	16 (26.7%)	NS
Abdominal bloating				
No	12 (21.8%)	32 (58.2%)	11 (20.0%)	
Yes	33 (27.0%)	62 (50.8%)	27 (22.1%)	NS
Food craving				
No	25 (22.1%)	63 (55.8%)	25 (22.1%)	
Yes	20 (31.2%)	31 (43.4%)	13 (20.3%)	NS
Change in appetite				
No	40 (25.0%)	85 (53.1%)	35 (21.9%)	
Yes	5 (29.4%)	9 (52.9%)	3 (17.6%)	NS

^a ^Data presented as numbers and percent within parenthesis.

## 4. Discussion

Premenstrual symptoms represent a wide group of physical and emotional symptoms of uncertain etiology and no definitive treatment that continues to adversely affect the quality of life and performance of a large segment of young women. These symptoms are highly underreported and years may pass before women seek medical help. The most notable finding of the current study is the very high incidence of premenstrual symptoms in young college students known with dysmenorrhoea (91.5%). The most common reported symptoms in our study of 177 young women were fatigue and mood swings at 72.9% and 72.3%, respectively. Thyse-Jacobs [27] in a review in 2000 hypothesized that disordered calciotropic hormone regulation is a major provocative factor in PMS. Calcium as a micronutrient is directly linked to the severity of the symptoms of PMS [28,29].Vitamin D and parathyroid hormone are key factors in calcium homeostasis. The issue of vitamin D and depression in general was discussed by Bertone-Johnson in 2009 [30] and the conclusion was that the evidence linking vitamin D and depression are largely circumstantial. However, low dietary vitamin D intake has been associated with the development of premenstrual symptoms [30]. In our study we found no difference in levels of vitamin D, whether normal, insufficient or deficient, between women with and those without premenstrual symptoms. Studies regarding vitamin D tested mostly the effect of supplements or dietary intake on premenstrual symptoms, where lower incidence is reported with high vitamin D intake [25,31]. This may not reflect contradictory results, however a variation in vitamin D utilization in women with and those without premenstrual symptoms reported by Tyse-Jacob in 2007 [32] suggested that variations in the levels of calcium regulating hormones all through the cycle are significant in patients with severe forms of PMS. As for parathyroid hormone, no difference in the incidence of hyperparathyroidism was detected in our subjects, while Thyse-Jacobs [33] linked transient secondary hyperparathyroidism to PMS. This difference in the results may reflect the difference in timing of blood sampling in our patients as compared to the Thyse-Jacobs study [33]. In our study the role of daily consumption of dairy products as a main source of calcium and other nutrients in relation to premenstrual symptoms failed to show any significant relation. Penland and Thyse-Jacobs [28,29] found that high calcium intake reduces the severity of premenstrual symptoms. As for each symptom, only headache and social withdrawal were significantly lower in those with high daily dairy product consumption. The strong relation between dysmenorrhoea and PMS may be related to disturbances in sex steroids production. 

Limitations: The diagnosis depends on recall of symptoms rather than a prospective symptom calendar and the percentage of spontaneous anovulatory cycles in dysmenorrheic women of this study was not determined due to logistic difficulties. In addition, the menstrual cycle phase of women was not determined when the blood samples were drawn

## 5. Conclusions

Premenstrual symptoms rates are very high among female college students who experience dysmenorrhea. No relation between premenstrual symptoms and Vitamin D, PTH or daily dairy products consumption was revealed in this study. There is a statistically significant negative relationship between dairy products consumption and headache and social withdrawal.
